# Influence of Radiation Dose to Reconstructed Breast Following Mastectomy on Complication in Breast Cancer Patients Undergoing Two-Stage Prosthetic Breast Reconstruction

**DOI:** 10.3389/fonc.2019.00243

**Published:** 2019-04-09

**Authors:** Jee Suk Chang, Seung Yong Song, Joo Hyun Oh, Dae Hyun Lew, Tai Suk Roh, Se Young Kim, Ki Chang Keum, Dong Won Lee, Yong Bae Kim

**Affiliations:** ^1^Department of Radiation Oncology, Yonsei Cancer Center, Yonsei University College of Medicine, Seoul, South Korea; ^2^Department of Plastic and Reconstructive Surgery, Severance Hospital, Yonsei University College of Medicine, Seoul, South Korea; ^3^Department of Plastic and Reconstructive Surgery, Gangnam Severance Hospital, Yonsei University College of Medicine, Seoul, South Korea

**Keywords:** breast reconstruction, mastectomy, implant, radiation dose, hypofractionated RT, dosimetric analysis

## Abstract

**Purpose:** This study investigated the association between radiation dose and complication rate in patients who underwent breast reconstruction to understand the role of radiation hypofractionated regimen, boost radiation therapy (RT), and RT techniques.

**Methods:** We retrospectively evaluated 75 patients treated with post-mastectomy adjuvant RT for breast cancer in the setting of two-stage prosthetic breast reconstruction. Near maximum radiation dose (D_max_) in the 2 or 0.03 cc of reconstructed breast or overlying breast skin was obtained from dose-volume histograms.

**Results:** Post-RT complications occurred in 22.7% of patients. Receiver operating characteristic analysis showed that all near D_max_ parameters were able to predict complication risk, which retained statistical significance after adjusting other variables (odds ratio 1.12 per Gy, 95% confidence interval 1.02–1.23) with positive dose-response relationship. In multiple linear regression model (*R*^2^ = 0.92), conventional fractionation (β = 11.7) and 16 fractions in 2.66 Gy regimen (β = 3.9) were the major determinants of near D_max_ compared with 15 fractions in 2.66 Gy regimen, followed by utilization of boost RT (β = 3.2). The effect of bolus and dose inhomogeneity seemed minor (*P* > 0.05). The location of hot spot was not close to the high density metal area of the expander, but close to the surrounding areas of partially deflated expander bag.

**Conclusions:** This study is the first to demonstrate a dose-response relationship between risk of complications and near D_max_, where hypofractionated regimen or boost RT can play an important role. Rigorous RT-quality assurance program and modification of dose constraints could be considered as a critically important component for ongoing trials of hypofractionation. Based on our findings, we initiated a multi-center retrospective study (KROG 18-04) and a prospective study (NCT03523078) to validate our findings.

## Introduction

Breast reconstruction provides important psychosocial, cosmetic, and quality of life benefits for women undergoing mastectomy (1, 2), which accounts for approximately more than 60% of mastectomy women in the US (3). As recent evidence has widened the indications of post-mastectomy radiation therapy (RT) to early-stage node-positive breast cancer (4), an increasing number of patients is currently referred for adjuvant RT to the reconstructed breast, which has put treating physicians in a challenging situation. There is substantial evidence from small case series and prospective cohort studies demonstrating that RT significantly increases complications following breast reconstruction regardless of the type of reconstructive surgery and timing of surgery (5, 6). Breasts reconstructed with implants are known to be more susceptible to RT-related complications compared with breasts reconstructed with autologous tissue (7). However, autologous approaches, which are resource- and labor-intensive and harbor a potential risk of acute morbidity, are not always feasible. Significant advances have been made in reconstructive surgical technique. Therefore, RT to prosthetic breast reconstruction is no longer contraindicated (3, 8).

RT has changed a little from 50 Gy for 5–6 weeks over the last several decades. Conventional planning for breast RT typically includes two tangential fields targeted to the whole breast or chest wall at an angle. Because of the conical shape of the breast, the radiation should traverse farther through the chest wall than the nipple areas, which inevitably causes significantly higher dose and dose inhomogeneity throughout the whole breast tissue. Because women's breasts differ in size and shape, the degree and location of “hot spot” doses in the breast are all different. In a recent retrospective study, researchers found that patients who developed reconstruction-related complications following post-mastectomy radiotherapy have higher degree of hot spots in breast skin than patients without complications, despite the same conventional RT technique at the same institution (9). This finding suggests a possible relationship between radiation hot spot doses and reconstruction-related complications, which can be more clinically relevant in contemporary practice where modulation of RT dose and homogeneity is available from dose/fractionation to treatment planning.

We hypothesized that the level of hot spot dose in skin or reconstructed-breast region might be associated with reconstruction-related complications in an independent manner. In the process of adopting hypofractionation for breast cancer at our institution between 2012 and 2016, three different dose/fraction schedules had been used with different RT delivery techniques with or without individualized boost irradiation to risky area, while surgical technique of two-stage prosthetic breast reconstruction was consistent among plastic surgeons during the same time period. In this context, we attempted to test our hypothesis in post-mastectomy patients who underwent RT to the breast with tissue expanders and planned to exchange the expander for a permanent implant afterwards.

## Materials and Methods

### Patients

Patients diagnosed with invasive breast cancer between January of 2012 and December of 2016 who underwent mastectomy with expander placement followed by post-mastectomy RT with intent to replace expander to implant-based reconstruction on a later date were included in this study. To control the possible effects of differing types of reconstruction and surgeon experience, this study was only limited to patients treated with immediate two-stage prosthetic reconstruction using acellular dermal matrix (ADM) by experienced plastic surgeons (DL and SS). During the study period, 1,552 patients underwent total mastectomy, 879 patients simultaneously underwent breast reconstruction and 504 patients subsequently received RT postoperatively at our institution. Patients with autologous reconstructions were excluded, yielding 75 patients. The study was approved by the Institutional Review Board of Severance Hospital, Yonsei University Health System.

### Breast Reconstruction

The first stage of the operation was performed simultaneously with the total unilateral mastectomy done by the oncologic surgery team. An expander was then inserted below the pectoralis muscle, slung with ADM to cover and reinforce the lower pole of the breast, and gradually inflated as much as possible. Inflation of expander started 2 weeks after the first operation, and the expander was partially deflated before post-mastectomy RT. After at least 3 months after completion of RT, the second stage of the operation including an exchange of expander to permanent implant was performed. In all cases, anatomical implants were used as a permanent implant.

### Radiation Therapy

RT was provided within 6 weeks after mastectomy or completion of the last cycle of adjuvant chemotherapy. All breast cancer patients underwent simulation computed tomography (CT) scans for RT planning. In cases with left-sided tumors and planned to undergo 3D-conformal RT, CT scans were acquired in both free breathing and deep inspiration breath hold phases using a respiration-monitoring device (Abches; APEX Medical, Inc., Tokyo, Japan) (10). The clinical target volume (CTV) including the ipsilateral chest wall, mastectomy scar, and regional nodal basins including axillary nodes, internal mammary lymph nodes, and supraclavicular lymph nodes was contoured in all patients according to the Radiation Therapy Oncology Group (RTOG) (11) or the European Society for Radiotherapy and Oncology (ESTRO) target volume guideline (12).

In conventional fractionation RT, chest wall was irradiated with two tangential photon beams and supraclavicular node with anterior photon beam with 50.4 Gy in 28 fractions. Field-in-field and wedge methods were used to improve dose homogeneity at the discretion of the treating physician. Bolus material was used in patients who have a high risk of skin recurrence to ensure that skin was covered adequately at the discretion of the physician.

In hypofractionated RT, the prescription dose was 40.05 Gy in 15 daily fractions of 2.67 Gy [UK START B regimen (13)] or 42.56 Gy in 16 daily fractions of 2.66 Gy [Canada Ontarian regimen (14)]. Bolus material was used in patients who had thin chest wall. The ventral border of chest wall CTV was moved 5 mm under the skin surface unless patients had T4 tumors. Use of arc-based intensity-modulated radiation therapy (IMRT) (Elekta Infinity Linac, Elekta, Crowley, UK) was recommended in hypofractionated schedule according to a national guideline for breast cancer radiation therapy.

At least 95% of the PTV should be covered by the 95% of the prescribed dose. Minimizing the occurrence of hot spot (Dmax [maximum point dose] < 105–107%) was strongly recommended particularly in chest wall skin PTV. The volume of ipsilateral lung at or exceeding 5 Gy (V5) and 20 Gy (V20) was mandated to be ≤ 45 and ≤ 20%, respectively, and mean heart dose to be >3 Gy in right-sided tumors and >5 Gy in left-sided tumors. Contralateral breast was constrained to be >1 Gy as a mean dose. Either concomitant or sequential boost radiation (6–10 Gy) was selectively used in patients with positive resection margin or residual or high-risk lymph node (e.g., internal mammary lymph nodal basin).

### Study Endpoints

The primary outcome measured was defined as reconstruction-related complication following reconstructive surgery at any time after the completion of post-mastectomy RT. Complications included capsular contracture, wound infection, wound dehiscence with implant exposure, and others, which required an unplanned operation or hospitalization. The complications were evaluated by two independent and blinded plastic surgeons. Various secondary outcomes measured were collected including radiation dermatitis based on Common Terminology Criteria for Adverse Events (version 4.0), survival, and recurrence outcomes.

### Analysis

**Figure 1:**
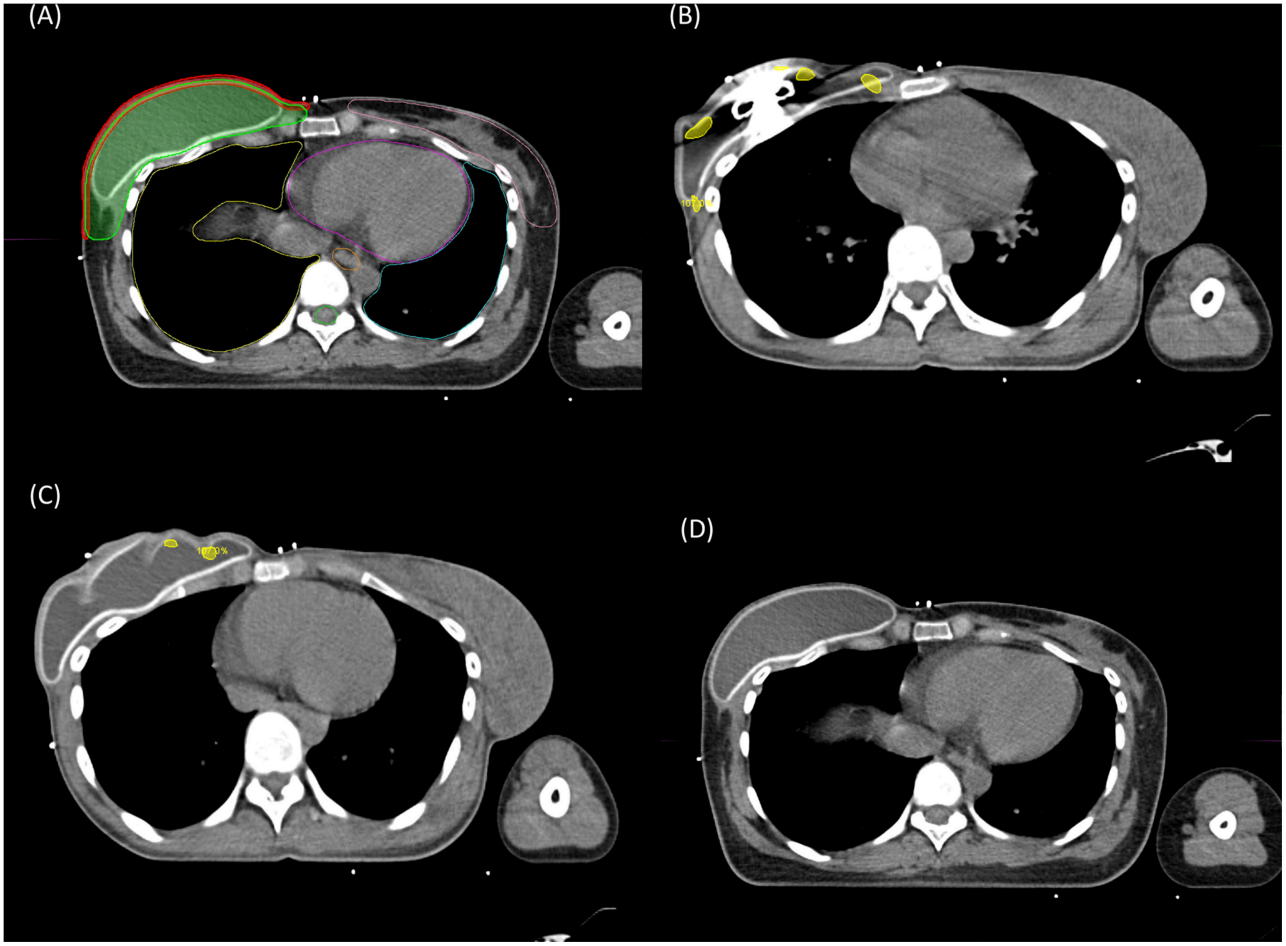
Contoured structures for reconstructed breast radiation therapy **(A)**. Red, 5 mm strip of the skin over the reconstructed breast; green, chest wall clinical target volume including the skin. The location of hot spot (107% of the prescribed dose) in partially deflated expander bag **(B,C)** and in fully inflated expander bag **(D)**.

Composite plans were created in MIM software (version 6.7.1; MIM Software Inc., Cleveland, OH, USA) by integrating all contours and dose distributions of patients in every RT session including baseline and adaptive cone down plans to display cumulative recalculated radiation dose. Skin volume was individually contoured as a 5 mm strip of skin over the tissue expander (Figure 1A) (15).

Near maximum dose (near D_max_) in the structure was calculated from 0.03 and 2 cc for regions of high absorbed dose (D_0.03cc_ and D_2cc_, respectively), rather than one single voxel point (point D_max_), as a more clinically relevant dose parameter. Near D_max_ of skin over the expander (skin near D_max_) and structure surrounding the entire expander (chest wall clinical target volume near D_max_) was obtained from dose-volume histograms.

The predictive values of each parameter for development of complication were tested using receiver operating characteristic (ROC) method. Logistic regression analysis was used to perform univariate and multivariate analyses and to analyze the dose-response relationship between the complication rate and dosimetric parameter. A multiple linear regression model was used to examine the effect of each factor on determining near D_max_. All statistical tests were two-sided, and a *P* < 0.05 was considered statistically significant. Statistical analyses were conducted using R software packages (http://www.r-project.org).

## Results

### Baseline Characteristics

A total of 75 patients were included. Baseline characteristics are summarized in Table 1. The average breast volume was 350 ± 136 ml. Seven percent of the patients had a history of diabetes. Four patients (5%) were former smokers, and no patient was current smoker at diagnosis. The median body mass index was 21.6 ± 2.9. A total of 38 (50.7%) patients had stage III disease, and 39 patients (52%) received neoadjuvant chemotherapy. In all patients, expander with ADM was inserted at the time of mastectomy. All patients received post-mastectomy RT to the chest wall and regional lymph nodes comprehensively including internal mammary node, axillary node, and supraclavicular lymph node. A bolus of 0.5 cm was applied in 25 patients (33.3%) in everyday settings until tolerated (median 37 Gy, range 18–50 Gy). Fifty patients received radiotherapy in hypofractionated schedules, and 25 patients received RT in conventional fractionation.

**Table 1:** Patient and treatment characteristics (*N* = 75).

		***N***	**%**
Age, years	Median (IQR)	40 (10)	
	≤40	39	52
	>40	36	48
BMI, kg/m^2^	Median (IQR)	21.6 (2.9)	
DM	Yes	5	7
Smoking history	Ex-smoker	4	5
	Current smoker	0	0
Histology	IDC	63	84
	Others	12	16
Laterality	Left	25	33
	Right	50	67
Bilateral disease	Bilateral	7	9
Final stage^*^	I	2	3
	IIA	16	21
	IIB	19	25
	IIIA	20	27
	IIIB	2	3
	IIIC	16	21
Grade	I	9	13
	II	47	65
	III	16	22
Molecular subtype	Luminal A	28	37
	Luminal B (HER2 negative)	18	24
	Luminal B (HER2 positive)	8	11
	HER2 positive (non-luminal)	9	12
	Triple negative	12	16
Breast volume (cc)	Left (median, IQR)	321 (205)	
	Right (median, IQR)	334 (207)	
RT Fraction schedule	267 cGy × 15	45	60
	267 cGy × 16	5	7
	180 cGy × 28 (200 cGy × 25)	25	33
RT plan	3D CRT	36	48
	VMAT	39	52
Boost RT	Yes	13	17
	Chest wall	5	
	IMN chain	7	
	SCL/AXL	1	
	No	62	83
Use of bolus material	Yes	25	33
	No	50	67
Neoadj chemo	No	36	48
	Anthracycline based	2	3
	Taxane based	26	35
	Taxane + HER2 directed therapy	11	15
Adj chemo	No	45	60
	Taxane non-containing	3	4
	Taxane containing	27	36
HER2-directed therapy^a^		17	100
Endocrine therapy^b^	No	1	2
	Tamoxifen	43	80
	Tamoxifen + LHRH agonist	6	11
	Aromatase inhibitor	4	7

*IQR, interquartile range; BMI, body mass index; DM, diabetes mellitus; IDC, invasive ductal carcinoma; HER2, human epidermal growth factor receptor 2; RT, radiation therapy; 3D CRT, three-dimensional conformal radiation therapy; VMAT, volumetric arc therapy; IMN, internal mammary node; SCL/AXL, supraclavicular/axillar lymph node, Neoadj, neoadjuvant; Adj, adjuvant; LHRH, Luteinizing hormone-releasing hormone*.

* *Higher of pathologic or prechemotherapy clinical stage*.

a *100% of patients with HER2-positive disease*.

b *100% of patients with hormone receptor-positive disease*.

### Acute Radiation Dermatitis

Grades 1 (faint erythema or dry desquamation), 2 (moderate to brisk erythema or patchy moist desquamation, mostly confined to skin folds), and 3 (moist desquamation, in areas other than skin folds) skin reaction occurred during or after RT in 33 (44%), 10 (13.3%), and 4 (5.3%) patients, respectively.

### Post-RT Reconstruction-Related Complication

Median follow-up was 32.5 months (range, 17.2–72.5 months); conventional RT vs. hypofractionated RT, 44 months (range, 17.6–72.5 months) vs. 30 months (range, 17.2–47.2 months, *P* = 0.002). Complications following reconstructive surgery at any time after the completion of RT occurred in 17 of 75 patients (22.7%). The complications in the conventional RT group included Baker grade III and IV capsular contractures (*n* = 8), wound dehiscence with implant exposure (*n* = 3), peri-prosthetic infection (*n* = 2), cellulitis (*n* = 1), and the conditions that required major revisional surgery such as coverage with latissimus dorsi flap (*n* = 2). The complications in the hypofractionated RT group included capsular contractures (*n* = 4) and cellulitis (*n* = 3). The mean time to complication from the date of RT completion was 8.1 ± 4.9 months. The complication rate was lower in patients with hypofractionated RT (14.3%) than in patients with conventional RT (38.5%, *P* = 0.017). A positive correlation was found between the severity of acute radiation dermatitis and complication rate [dermatitis G0 3/28 (10.7%), G1 5/33 (15.2%), G2 7/10 (70%), and G3 2/4 (50%), *P* < 0.001].

### Dosimetric Analysis

**Figure 2:**
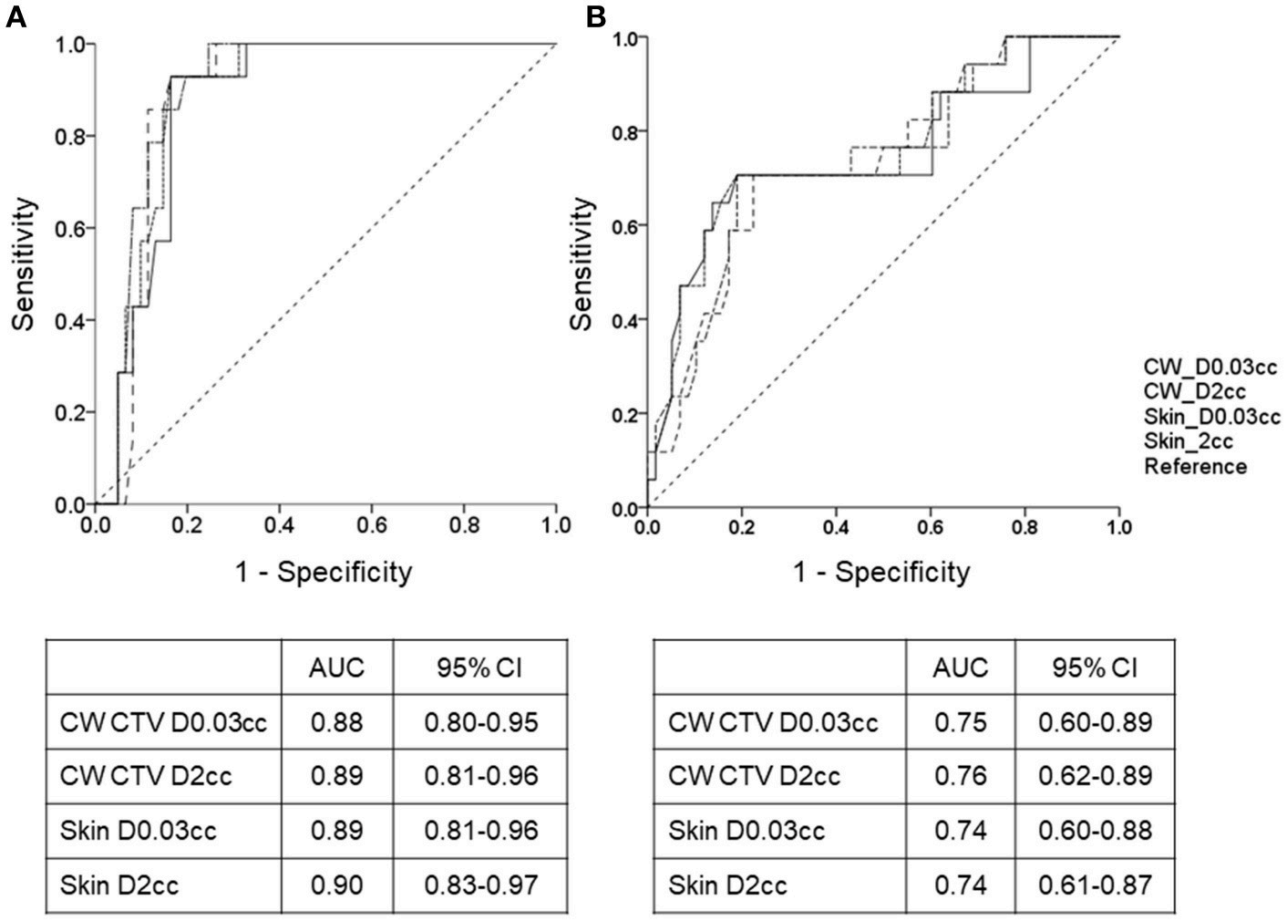
Receiver operating characteristic (ROC) curve and comparison of dosimetric parameters for development of **(A)** grade 2+ radiation dermatitis and **(B)** post-RT reconstruction-related complication between the areas under the ROC curve. AUC, area under curve; CW, chest wall; CI, confidence interval.

ROC analysis showed that “near D_max_,” such as doses to the hottest 0.03 and 2 cc of chest wall clinical target volume and skin, was able to predict development of both grade ≥2 acute radiation dermatitis and post-RT reconstruction complication (Figure 2). To test the robustness of this finding, ROC analyses were re-performed after conversion to an equivalent dose in 2-Gy fractions (α/β = 10 or 3) considering the various fractionation schedules used. Similar results have been observed regardless of dose conversion (Supplementary Figure 1).

**Figure 3:**
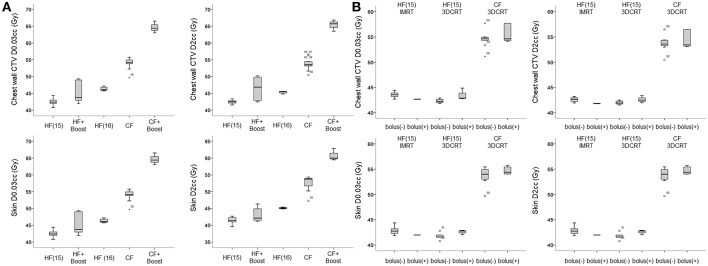
**(A)** Near D_max_ values according to fractionation regimen and use of boost RT. HF (15), 15 fractions in 2.66 Gy; HF (16), 16 fractions in 2.66 Gy; CF, conventional fractionation. **(B)** Near D_max_ values according to RT techniques and use of bolus material. CTV, clinical target volume; IMRT, intensity-modulated RT; 3DCRT, 3-dimensional conformal RT.

**Figure 4:**
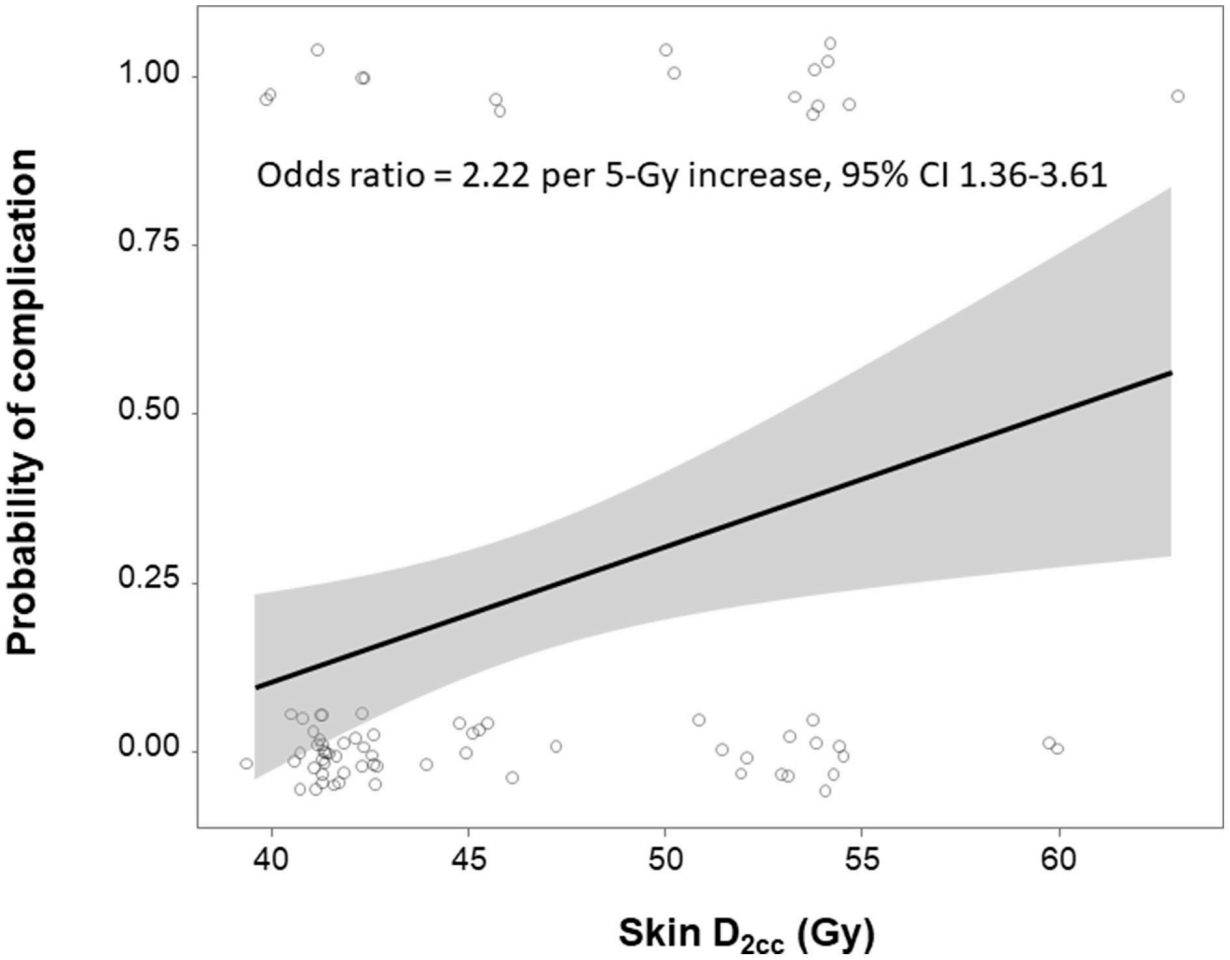
Dose-response relationship between skin D_2cc_ and development of reconstruction-related complication. Shaded gray regions indicate the 95% confidence interval.

Near D_max_ (skin D_2cc_) retained statistical significance after adjusting other variables (odds ratio 1.12; Table 2). Conventional fractionation regimen (control, 2.66 Gy × 15 fraction regimens; β = 11.7) was a major determinant of near D_max_ level, followed by 2.66 Gy × 16 fraction hypofractionation regimens (control, 2.66 Gy × 15 fraction regimens; β = 3.9), and use of boost RT (β = 3.2, *R*^2^ = 0.92, *P* < 0.001, Table 3). Using these three factors, the patients can be sorted in ascending order by near D_max_ between 40 and 70 Gy (Figure 3A). Although use of IMRT or tissue-equivalent bolus material did not significantly influence on the hottest dose level in the multiple linear regression model, use of bolus changed the near D_max_ level ~1–3 Gy in 3-dimensional conformal RT (3DCRT) patients, but not in IMRT patients, under the same condition of fractionation regimen (Figure 3B). The dose-response relationship between reconstruction-related complication and skin D_2cc_ was analyzed and the probability increased as the dose increased (Figure 4). The location of the radiation hot spot was investigated, and it was not close to the high density metal area of the expander but close to the surrounding areas of partially deflated expander bag (Figures 1B–D).

**Table 2:** Unadjusted and adjusted odds ratios for association with reconstruction-related complications for each patient and treatment characteristic.

		**Univariable**			**Multivariable**		
		**OR**	**95% CI**	***P***	**OR**	**95% CI**	***P***
Age, year	(Continuous)	0.98	0.92–1.04	0.533	1.01	0.94–1.07	0.857
Body mass index, kg/m^2^	(Continuous)	0.84	0.67–1.06	0.143	0.81	0.61–1.07	0.138
Smoking history	Yes vs. No	1.15	0.11–11.8	0.909	0.6	0.05–7.02	0.688
Skin D2cc, Gy	(Continuous)	1.11	1.02–1.22	0.018	1.12	1.02–1.23	0.015

*OR, odds ratio; CI, confidence interval; D2cc, maximum dose in the most exposed tissues of the skin (2 cc)*.

**Table 3:** Coefficients entered in multiple linear regression model for radiation therapy variables and dose in the most-exposed 2 cc of skin (D2cc).

**Variable entered in model**	**ß coefficient**	**SE**	***P*-value**
180 cGy × 28 (200 cGy × 25) vs. 267 cGy × 15	11.73	0.59	<0.001
267 cGy × 16 vs. 267 cGy × 15	3.85	0.9	<0.001
Use of boost RT^a^	3.20	0.57	<0.001
3DCRT vs. IMRT	0.86	0.58	0.138
Use of bolus material	0.13	0.48	0.790
Intercept	40.13	0.79	
Adjusted *R*^2^	0.92		<0.001

*SE, standard error; 3DCRT, three-dimensional conformal radiation therapy; IMRT, intensity-modulated radiation therapy*.

a *Chest wall boost (n = 5), internal mammary node boost (n = 7), and supraclavicular node boost (n = 1)*.

### Recurrence

During the follow-up period, no local recurrence was found in the chest wall. There were 7 distant metastases with simultaneous regional recurrence (*n* = 1) and 2 cancer-related deaths. The 3-year overall and disease-free survival rates were 97.1 and 89.2%, respectively. The 3-year disease-free survival rates were 82.6% (95% CI, 66.7–98.5%) and 93.8% (95% CI, 86.9–100%) in the conventional RT and hypofractionated RT groups, respectively.

## Discussion

Although there have been outstanding advances in reconstructive procedures and materials, post-mastectomy RT remains to have a profound impact on complications specifically related to reconstruction (16). In post-mastectomy setting, there was not much to change regarding RT regimen and techniques (which govern the dose homogeneity), since RT dose and techniques have not changed greatly and fixed on 25 fractions of 2 Gy per fraction over the last several decades.

The present retrospective analysis included 75 patients with breast cancer treated with two-stage prosthetic breast reconstruction and post-operative radiation. As mentioned in the Introduction section, a consistent discrepancy exists regarding RT technique and fractionation schedule among physicians in our institution from 2012 to 2016. Some physicians prefer conventional fractionation regimen with standard tangent techniques, whereas others prefer hypofractionated regimen (15–16 fractions of 2.66 Gy) with IMRT technique. As a result, the present study included patients treated with a wide range of radiation dose, which allows to analyze the association of radiation dose with complication risk. We found a spectrum of post-RT reconstruction-related complications depending on near D_max_ in the reconstructed breast.

This finding is in line with recent observations by Muresan et al. from 83 patients with implant-based reconstructions showing that increased maximum skin dose (D_1cc_) is associated with complications (9). Prone positioning technique was suggested to be used to decrease the maximum skin dose than supine positioning (58.5 Gy vs. 61.7 Gy, *P* < 0.001). Given that radiation hot spot level is determined by the combination of prescribed radiation dose and dose inhomogeneity, the present study discovered the former aspect to complication, and the study of Muresan et al. highlighted the latter aspect.

The rationale for hypofractionated regimen stemmed from the emerging evidence that breast cancer cells are more sensitive to increased daily fraction size. Multiple randomized trials investigated progressively more condensed regimens (15–16 fractions of 2.66 Gy 13 → fractions of 3/3.2/3.33 Gy → 5 fractions of 5.2–5.4 Gy) for breast cancer (mostly in patients with intact breast who underwent breast conservation therapies) (17). All hypofractionated regimens have equivalent local control effect to conventional fractionation regimen (18–21). Based on these data, the ASTRO guideline has been recently updated to expand the population of patients recommended to receive hypofractionated regimen (22). Nonetheless, hypofractionated regimens have been adopted slowly in Korea and the United States where fear of large daily fraction dose is deep-seated and fee-for-service is used (23, 24).

Data supporting hypofractionated RT after mastectomy are limited, especially if accompanied by breast reconstruction. The transformative effect of hypofractionated RT in patients with breast conservation therapies has also accelerated investigation of hypofractionated RT in the setting of mastectomy with or without reconstruction. In 2017, the study of Khan et al. involving 69 patients with stage II to IIIa reported promising results of hypofractionated post-mastectomy radiotherapy using standard tangent techniques (11 fractions of 3.33 Gy) with low toxicity and high local control (20). Reconstruction was performed in 41 patients, and an overall grade 3 or more reconstruction toxicity of 32% was observed, which is in line with previously reported rates. Another 2009 study from the United Kingdom, where hypofractionation has been commonplace, compared the risk of capsular contracture between 41 women receiving hypofractionation (15 factions of 2.66 Gy) and 137 without RT in the setting of implant-based reconstruction (25). The toxicity rate was significantly higher in RT arm for a crude rate of 19.5% and up to 30% at 5 years. In 2019, a randomized, phase 3 trial in China found the equivalent efficacy and toxicity of 43.5 Gy in 15 fractions of hypofractionated RT compared with conventional treatment in patients with mastectomy without breast reconstruction (26).

A variety of hypofractionated regimens has been tested worldwide. Some regimens, such as 13 fractions of 3.3 Gy, where total dose is greater than conventional fractionation regimen when converted into biological equivalent dose (BED), were associated with more breast shrinkage, distortion, and induration, among women irradiated to the intact breast. Other regimens, such as 15 fractions of 2.66 Gy, which is expected to have lower BED than conventional fractionation, were associated with less induration, shrinkage, and edema. We identified a positive dose-response relationship between reconstruction-related complication and radiation dose (adjusted odds ratio, 2.22 per 5 Gy increase; 95% CI 1.36–3.61), and the different hypofractionation regimens were found to have greatest impact on the degree of radiation dose. Individualized boost RT (median 8 Gy) was used in 17% of our patients to treat microscopic tumor cells in localized high-risk area. Use of boost RT contributed to increase radiation hottest dose as the third priority in multiple linear regression analysis (β = 3.20).

In contrast to the study by Muresan et al. we did not find a significant difference in hot point dose by different RT techniques because the hottest dose was strictly restricted under 105–107% of prescription dose regardless of RT technique according to institutional dose constraint policy. Even in patients treated with standard tangent techniques, field-in-field or wedge technique was used to improve dose homogeneity. Upcoming randomized trials of hypofractionation (Alliance A221505, NCT03414970, and FABREC, NCT03422003) are initiated to compare the complication rate or patient-reported outcomes of hypofractionated RT and conventional RT in mastectomy patients with reconstruction. However, considering our findings, there is an increasing concern about whether possible confounders will be adequately controlled to compare two schemes, because in an earlier phase II study (20), a maximum prescription dose of 120% was allowed within the limit of 2 cc, which contains a risk of higher maximum point dose more than 120%. Therefore, dose homogeneity, fractionation regimen, and use of boost RT should be considered in clinical trials investigating the RT effect on reconstruction.

Unlike RT to intact breast after breast conservation surgery, skin is generally included to radiation target in the post-mastectomy RT setting. Although a tissue-equivalent bolus material is generally used to increase skin dose by overcoming the build-up phenomenon, the use of bolus material did not significantly increase the hottest dose in the skin, which is estimated from the radiation treatment planning system. Considering that 1–3 Gy variation was observed in patients treated with 3DCRT, but not in IMRT, the discrepancy can be explained by a large proportion (52%) of IMRT delivery in the present cohort because dose to skin is more dependent on target volume contouring whether the ventral border of breast target volume delineated 3–5 mm under the skin surface or not.

In the present study, severity of RT-related dermatitis significantly correlated with post-RT complication risk (G0-1 vs. G2+; 13.1% vs. 64.3%, *P* < 0.001), which provided indirect evidence of dose-response relationship hypothesis. This is explicitly in line with the study by Parsa et al. that acute RT-related change could be a valid predictive marker for modified Baker grade IV capsular contracture in 27 patients who were undergoing delayed expander-implant reconstruction (27). Poor cosmetic outcomes were observed after reconstruction in more than three-fourths of the 15 patients who developed severe skin changes or induration by RT. However, no poor aesthetic outcome was found in 27 patients with non-irradiated chest walls and in 12 patients who received irradiation to chest wall but had no induration and moderate skin changes. Considering that several factors including patient's scarring tendency and a chronic inflammatory process contribute to the development of reconstruction complication, such as capsular contracture (28), radiation is well-known to induce inflammation so that the severity of radiation dermatitis can be a good surrogate of post-RT complication risk.

The second indirect evidence of our hypothesis is that the location of the hottest radiation dose area was close to the crumpled area of the deflated expander bag in patients whose expander bag was partially deflated immediately before RT simulation (Figures 1B–D). It avoids the possibility of calculation artifact in the planning system, but implies a true “hot spot” region in tissue. No international consensus on expander inflation/deflation status during RT has been established. Details of RT for patients who underwent mastectomy and breast reconstruction vary widely, according to a recent nationwide survey in the US and Korea (8) (Chang et al., manuscript in process). A majority of patients in Korea (89%) had deflated expander bag prior to radiation in a greater or lesser extent, whereas 75.2% of physicians in the US responded that they do not routinely deflate the expander bag. A recent retrospective study of 49 patients in Korea found that RT-related complications are significantly reduced in the maximal inflation group (29). In this context, we changed our practice to maintain maximal inflation at the time of RT, but this should be a subject of an ongoing research.

This study has limitations inherent to any retrospective studies. Effects of bolus and dose inhomogeneity on complication rates were not observed in this study, however the analysis is probably limited in this study cohort, where the maximum dose was consistently controlled below 105–107%. Another caveat is that implant size elected by Korean women are generally smaller than their American counterparts similarly Korean women have a lower body mass index than American women. Selection bias, different median follow-up times between hypofractionated and conventional fractionated RT, and unmeasured confounding possibly existed and yielded exacerbated risk in the conventional RT group. Since the actual rate of reconstruction-related complications in this study is quite similar to that in previous studies, we do not expect that it actually occurred. A longer follow-up is necessary to estimate accurate complication rates because it increases as the duration of patient follow-up increases. Moreover, we attempted to adequately control surgical confounders of complication by limiting the study eligibility to patients who underwent the same procedures with ADM by two experienced surgeons.

To our knowledge, this study is the first to demonstrate radiation dose-response and complications, as well as the effect of hypofractionated regimens and other radiation factors including use of boost RT, RT technique, and tissue-equivalent bolus. As a radiation technique, fractionation schedule, planning, and delivery evolve, a number of factors increase the influence of near D_max_ on complications. Efforts to decrease radiation dose by focusing either on selecting hypofractionated regimen or modifying boost RT are likely to have a major effect. The finding that multiple hypofractionated regimens yielded similar treatment outcomes and late toxicity but varied acute toxicity according to different regimens in patients with intact breast after breast conservation surgery suggest that some hypofractionated regimens have the potential to reduce complications in the setting of breast reconstruction. Our results are hypothesis generating, and confirmatory studies with external datasets are mandatory. However, these results can provide a foundation for future protocols to improve the outcomes of breast reconstruction in post-mastectomy RT setting. On the basis of our findings, we conducted multi-center retrospective studies to validate our findings (KROG 18-04). We also initiated a prospective multi-institutional study to evaluate patient-reported outcomes in patients treated with breast reconstruction and different fractionation regimens (NCT 03523078). For ongoing and future trials, rigorous dose quality assurance and modification of dose constraints could be considered as a critically important component.

## Author Contributions

JC, DWL, and YK drafted the manuscript and worked on the conception, design, and interpretation of data. JC, JO, SS, DWL, and YK performed the data analysis. JC, JO, SS, DHL, TR, SK, KK, DWL, and YK reviewed the data analysis and study conclusions. All authors approved the final version of the manuscript.

### Conflict of Interest Statement

The authors declare that the research was conducted in the absence of any commercial or financial relationships that could be construed as a potential conflict of interest.

## Supplementary Material

Supplementary Figure 1 Receiver operating characteristic (ROC) curve and comparison of dosimetric parameters for development of post-RT reconstruction-related complication between the areas under the ROC curve after conversion to an equivalent dose in 2-Gy fractions [α/β = 3 **(A)** or 10 **(B)**]. AUC, area under curve; CW CTV, chest wall clinical target volume; CI, confidence interval.

## References

[1] Susarla SM Ganske I Helliwell L Morris D Eriksson E Chun YS. Comparison of clinical outcomes and patient satisfaction in immediate single-stage versus two-stage implant-based breast reconstruction. Plast Reconstr Surg. (2015) 135:1e−8e. 10.1097/PRS.0000000000000803 25539329

[2] Jagsi R Jiang J Momoh AO Alderman A Giordano SH Buchholz TA. Complications after mastectomy and immediate breast reconstruction for breast cancer: a claims-based analysis. Ann Surg. (2016) 263:219–27. 10.1097/SLA.0000000000001177 25876011 PMC4824182

[3] Jagsi R Jiang J Momoh AO Alderman A Giordano SH Buchholz TA. Trends and variation in use of breast reconstruction in patients with breast cancer undergoing mastectomy in the United States. J Clin Oncol. (2014) 32:919–26. 10.1200/JCO.2013.52.2284 24550418 PMC4876312

[4] EBCTCG (Early Breast Cancer Trialists' Collaborative Group) Effect of radiotherapy after mastectomy and axillary surgery on 10-year recurrence and 20-year breast cancer mortality: meta-analysis of individual patient data for 8135 women in 22 randomised trials. Lancet. (2014) 383:2127–35. 10.1016/S0140-6736(14)60488-8 24656685 PMC5015598

[5] Ho A Cordeiro P Disa J Mehrara B Wright J Van Zee KJ. Long-term outcomes in breast cancer patients undergoing immediate 2-stage expander/implant reconstruction and postmastectomy radiation. Cancer. (2012) 118:2552–9. 10.1002/cncr.26521 21918963

[6] Ayoub Z Strom EA Ovalle V Perkins GH Woodward WA Tereffe W. A 10-year experience with mastectomy and tissue expander placement to facilitate subsequent radiation and reconstruction. Ann Surg Oncol. (2017) 24:2965–71. 10.1245/s10434-017-5956-6 28766219

[7] Kronowitz SJ. Current status of implant-based breast reconstruction in patients receiving postmastectomy radiation therapy. Plast Reconstr Surg. (2012) 130:513e−23e. 10.1097/PRS.0b013e318262f059 23018711 PMC3898175

[8] Thomas K Rahimi A Spangler A Anderson J Garwood D. Radiation practice patterns among United States radiation oncologists for postmastectomy breast reconstruction and oncoplastic breast reduction. Pract Radiat Oncol. (2014) 4:466–71. 10.1016/j.prro.2014.04.002 25407870

[9] Muresan H Lam G Cooper BT Perez CA Hazen A Levine JP. Impact of evolving radiation therapy techniques on implant-based breast reconstruction. Plast Reconstr Surg. (2017) 139:1232e−9e. 10.1097/PRS.0000000000003341 28538549

[10] Lee HY Chang JS Lee IJ Park K Kim YB Suh CO. The deep inspiration breath hold technique using Abches reduces cardiac dose in patients undergoing left-sided breast irradiation. Radiat Oncol J. (2013) 31:239–46. 10.3857/roj.2013.31.4.239 24501713 PMC3912239

[11] White J Tai A Arthur D Buchholz T MacDonald S Marks L Breast Cancer Atlas for Radiation Therapy Planning: Consensus Definitions. Available online at: https://www.rtog.org/LinkClick.aspx?fileticket=vzJFhPaBipE (accessed August 31, 2017).

[12] Offersen BV Boersma LJ Kirkove C Hol S Aznar MC Biete Sola A. ESTRO consensus guideline on target volume delineation for elective radiation therapy of early stage breast cancer. Radiother Oncol. (2015) 114:3–10. 10.1016/j.radonc.2014.11.030 25630428

[13] Group ST Bentzen SM Agrawal RK Aird EGA Barrett JM Barrett-Lee PJ The UK Standardisation of Breast Radiotherapy (START) trial B of radiotherapy hypofractionation for treatment of early breast cancer: a randomised trial. Lancet. (2008) 371:1098–107. 10.1016/S0140-6736(08)60348-7 18355913 PMC2277488

[14] Whelan TJ Pignol J-P Levine MN Julian JA MacKenzie R Parpia S. Long-term results of hypofractionated radiation therapy for breast cancer. N Engl J Med. (2010) 362:513–20. 10.1056/NEJMoa0906260 20147717

[15] Capelle L Warkentin H MacKenzie M Joseph K Gabos Z Pervez N. Skin-sparing helical tomotherapy vs. 3D-conformal radiotherapy for adjuvant breast radiotherapy: *in vivo* Skin Dosimetry study. Int J Radiat Oncol Biol Phys. (2012) 83:e583–90. 10.1016/j.ijrobp.2012.01.086 22580119

[16] Clemens MW Kronowitz SJ. Current perspectives on radiation therapy in autologous and prosthetic breast reconstruction. Gland Surg. (2015) 4:222–31. 10.3978/j.issn.2227-684X.2015.04.03 26161307 PMC4461707

[17] Valle LF Agarwal S Bickel KE Herchek HA Nalepinski DC Kapadia NS. Hypofractionated whole breast radiotherapy in breast conservation for early-stage breast cancer: a systematic review and meta-analysis of randomized trials. Breast Cancer Res Treat. (2017) 162:409–17. 10.1007/s10549-017-4118-7 28160158

[18] Group ST Bentzen SM Agrawal RK Aird EG Barrett JM Barrett-Lee PJ The UK Standardisation of Breast Radiotherapy (START) trial A of radiotherapy hypofractionation for treatment of early breast cancer: a randomised trial. Lancet Oncol. (2008) 9:331–41. 10.1016/S1470-2045(08)70077-9 18356109 PMC2323709

[19] Jagsi R Griffith KA Boike TP Walker E Nurushev T Grills IS. Differences in the acute toxic effects of breast radiotherapy by fractionation schedule: comparative analysis of physician-assessed and patient-reported outcomes in a large multicenter cohort. JAMA Oncol. (2015) 1:918–30. 10.1001/jamaoncol.2015.2590 26247417

[20] Khan AJ Poppe MM Goyal S Kokeny KE Kearney T Kirstein L. Hypofractionated postmastectomy radiation therapy is safe and effective: first results from a prospective phase II trial. J Clin Oncol. (2017) 35:2037–43. 10.1200/JCO.2016.70.7158 28459606 PMC5476174

[21] Sun GY Wang SL Song YW Jin J Wang WH Hypofractionated radiotherapy after mastectomy for the treatment of high-risk breast cancer: 5-year follow up result of a randomized trial [abstract]. Int J Radiat Oncol Biol Phys. (2017) 99:S3–4. 10.1016/j.ijrobp.2017.06.024

[22] Smith BD Bellon JR Blitzblau R Freedman G Haffty B Hahn C. Radiation therapy for the whole breast: executive summary of an American Society for Radiation Oncology (ASTRO) evidence-based guideline. Pract Radiat Oncol. (2018) 8:145–52. 10.1016/j.prro.2018.01.012 29545124

[23] Jagsi R Falchook AD Hendrix LH Curry H Chen RC. Adoption of hypofractionated radiation therapy for breast cancer after publication of randomized trials. Int J Radiat Oncol Biol Phys. (2014) 90:1001–9. 10.1016/j.ijrobp.2014.09.032 25539365

[24] Park HJ Oh DH Shin KH Kim JH Choi DH Park W. Patterns of practice in radiotherapy for breast cancer in Korea. J Breast Cancer. (2018) 21:244–50. 10.4048/jbc.2018.21.e37 30275852 PMC6158163

[25] Whitfield GA Horan G Irwin MS Malata CM Wishart GC Wilson CB. Incidence of severe capsular contracture following implant-based immediate breast reconstruction with or without postoperative chest wall radiotherapy using 40 Gray in 15 fractions. Radiother Oncol. (2009) 90:141–7. 10.1016/j.radonc.2008.09.023 18977547

[26] Wang S-L Fang H Song Y-W Wang W-H Hu C Liu Y-P. Hypofractionated versus conventional fractionated postmastectomy radiotherapy for patients with high-risk breast cancer: a randomised, non-inferiority, open-label, phase 3 trial. Lancet Oncol. (2019) 20:352–60. 10.1016/S1470-2045(18)30813-1 30711522

[27] Parsa AA Jackowe DJ Johnson EW Lye KD Iwahira Y Huynh TV. Selection criteria for expander/implant breast reconstruction following radiation therapy. Hawaii Med J. (2009) 68:66–8. Available online at: https://www.hjmph.org/HMJ_Apr09.pdf#page=19 19441617

[28] Araco A Caruso R Araco F Overton J Gravante G. Capsular contractures: a systematic review. Plast Reconstr Surg. (2009) 124:1808–19. 10.1097/PRS.0b013e3181bf7f26 19952637

[29] Woo KJ Paik JM Bang SI Mun GH Pyon JK. The impact of expander inflation/deflation status during adjuvant radiotherapy on the complications of immediate two-stage breast reconstruction. Aesthetic Plast Surg. (2017) 41:551–9. 10.1007/s00266-017-0864-5 28374300

