# Supplementary figures and images for: Influence of Radiation Dose to Reconstructed Breast Following Mastectomy on Complication in Breast Cancer Patients Undergoing Two-Stage Prosthetic Breast Reconstruction
Source: Front Oncol. 2019 Apr 9;9:243. doi: 10.3389/fonc.2019.00243 (PMC6465567; doi:10.3389/fonc.2019.00243)

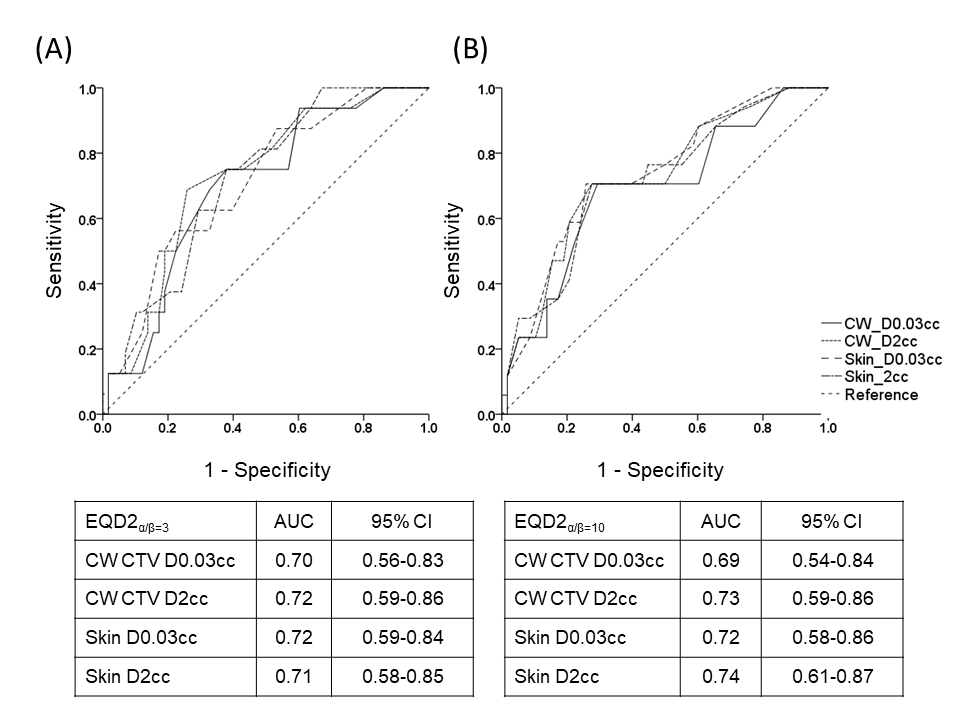

Supplement: Supplementary Figure 1 — Receiver operating characteristic (ROC) curve and comparison of dosimetric parameters for development of post-RT reconstruction-related complication between the areas under the ROC curve after conversion to an equivalent dose in 2-Gy fractions [α/β = 3 (A) or 10 (B)]. AUC, area under curve; CW CTV, chest wall clinical target volume; CI, confidence interval. [file Image_1.TIF]
